# High mobility group box 1 levels in large vessel vasculitis are not associated with disease activity but are influenced by age and statins

**DOI:** 10.1186/s13075-015-0672-8

**Published:** 2015-06-12

**Authors:** Alexandre W. S. de Souza, Kornelis S. M. van der Geest, Elisabeth Brouwer, Frederico A. G. Pinheiro, Ana Cecília Diniz Oliveira, Emília Inoue Sato, Luis Eduardo C. Andrade, Marc Bijl, Johanna Westra, Cees G. M. Kallenberg

**Affiliations:** Department of Rheumatology and Clinical Immunology, University of Groningen, University Medical Center Groningen, Hanzeplein 1, 9700 RB Groningen, The Netherlands; Rheumatology Division, Universidade Federal de São Paulo – Escola Paulista de Medicina, R. Botucatu, 720, 04023 900 São Paulo, SP Brazil; Department of Internal Medicine and Rheumatology, Martini Hospital, Van Swietenplein 1, 9728 NT Groningen, The Netherlands

## Abstract

**Introduction:**

Takayasu arteritis (TA) and giant cell arteritis (GCA) are large vessel vasculitides (LVV) that usually present as granulomatous inflammation in arterial walls. High mobility group box 1 (HMGB1) is a nuclear protein that acts as an alarmin when released by dying or activated cells. This study aims to evaluate whether serum HMGB1 can be used as a biomarker in LVV.

**Methods:**

Twenty-nine consecutive TA patients with 29 healthy controls (HC) were evaluated in a cross-sectional study. Eighteen consecutive GCA patients with 16 HC were evaluated at the onset of disease and some of them during follow-up. Serum HMGB1 levels were measured by enzyme-linked immunosorbent assay.

**Results:**

In GCA patients at disease onset mean serum HMGB1 levels did not differ from HC (5.74 ± 4.19 ng/ml vs. 4.17 ± 3.14 ng/ml; *p* = 0.230). No differences in HMGB1 levels were found between GCA patients with and without polymyalgia rheumatica (*p* = 0.167), ischemic manifestations (*p* = 0.873), systemic manifestations (*p* = 0.474) or relapsing disease (*p* = 0.608). During follow-up, no significant fluctuations on serum HMGB1 levels were observed from baseline to 3 months (*n* = 13) (*p* = 0.075), 12 months (*n* = 6) (*p* = 0.093) and at the first relapse (*n* = 4) (*p* = 0.202). Serum HMGB1 levels did not differ between TA patients and HC [1.19 (0.45–2.10) ng/ml vs. 1.46 (0.89–3.34) ng/ml; *p* = 0.181] and no difference was found between TA patients with active disease and in remission [1.31 (0.63–2.16) ng/ml vs. 0.75 (0.39–2.05) ng/ml; *p* = 0.281]. HMGB1 levels were significantly lower in 16 TA patients on statins compared with 13 patients without statins [0.59 (0.29–1.46) ng/ml vs. 1.93 (0.88–3.34) ng/ml; *p* = 0.019]. Age was independently associated with higher HMGB1 levels regardless of LVV or control status.

**Conclusions:**

Patients with TA and GCA present similar serum HMGB1 levels compared with HC. Serum HMGB1 is not useful to discriminate between active disease and remission. In TA, use of statins was associated with lower HMGB1 levels. HMGB1 is not a biomarker for LVV.

## Introduction

Takayasu arteritis (TA) and giant cell arteritis (GCA) are large vessel vasculitides (LVV) characterized by granulomatous inflammation of the vessel wall [1]. Although both diseases present significant overlap in features and some similarities in the distribution of angiographic lesions [2], TA predominantly affects young females and involves the aorta and its main branches whereas GCA affects predominantly branches of carotid and vertebral arteries in individuals older than 50 years [1].

Despite clinical symptoms, acute phase reactants and vascular imaging help to assess disease activity in LVV, there is a need for novel biomarkers for diagnosis, prognosis and to distinguish active disease from damage or infection. In TA, active disease is associated with higher serum levels of pentraxin-3, matrix metalloproteinase 9 (MMP-9), interleukin (IL)-6, IL-8, IL-18, B cell-activating factor (BAFF), monocyte chemoattractant protein-1 (MCP-1) and regulated on activation, normal T cell expressed and secreted (RANTES) [3–9]. In GCA, high serum levels of tumor necrosis factor alpha (TNF-α), IL-6, IL-10, chemokine (C-X-C motif) ligand 9 (CXCL9) and BAFF are associated with active disease while serum levels of CC chemokines CCL2 and CCL11 are decreased at disease onset [10–14]. Moreover, adaptive immunity is triggered during GCA pathogenesis manifested by T helper (Th)1 and Th17 responses with the production of interferon (IFN)-γ and IL-17A, which enhance arterial inflammation [15, 16].

High mobility group box 1 (HMGB1) is a nuclear nonhistone protein that acts as an alarmin when released into the extracellular milieu either by cellular death or upon activation of inflammatory cells, e.g. macrophages by lipopolysaccharide (LPS) or IFN-γ [17, 18]. High serum HMGB1 levels have been observed in infectious diseases, atherosclerosis, mechanical trauma, cancer, and in systemic autoimmune diseases such as systemic lupus erythematosus (SLE) [19–23]. In systemic vasculitis, high serum HMGB1 levels were observed in Kawasaki disease, immunoglobulin (Ig)A vasculitis, and in patients with antineutrophil cytoplasmic antibody (ANCA)-associated vasculitis, especially in granulomatosis with polyangiitis (GPA) with granulomatous manifestations [24–27]. Serum HMGB1 levels have not been evaluated in patients with LVV. This study aims to evaluate serum HMGB1 levels as a surrogate marker of disease activity in patients with LVV and associations between serum HMGB1 and acute phase reactants, disease manifestations and therapy in patients with TA and GCA. Due to epidemiological differences in the prevalence of both diseases, patients with TA were recruited from Brazil whereas GCA patients were recruited from The Netherlands.

## Methods

### Study population

The study comprised 18 GCA patients with 16 healthy controls (HC), both from the University Medical Center Groningen (UMCG), The Netherlands (Table 1), and 29 consecutive TA patients from Universidade Federal de São Paulo (UNIFESP), Brazil with 29 HC from the same region (Table 1). Inclusion criterion for TA patients was the fulfillment of the 1990 American College of Rheumatology (ACR) classification criteria [28] while the exclusion criteria were current chronic infectious disease, malignancy, and pregnancy. GCA patients were included if they fulfilled the 1990 ACR criteria [29] or when presenting compatible manifestations associated with an enhanced 18^F^-fluorodeoxyglucose uptake in large vessels by positron emission computed tomography (18FDG-PET/CT). Exclusion criteria for GCA included current chronic infectious disease and malignancy. The study was approved by the Ethics Committee on Research from UNIFESP and by the Medical Ethical Committee of UMCG and complied with the Declaration of Helsinki. All necessary consent was provided from all participants involved in this study.

**Table 1:** Demographic, disease features and therapy of patients with giant cell arteritis at disease onset and Takayasu arteritis

Variables	GCA	HC	*p*	Variables	TA	HC	*p*
	(*n* = 18)	(*n* = 16)			(*n* = 29)	(*n* = 29)	
Demographic features							
Age, years	72.0 (63.7–75.0)	68.5 (63.0–72.0)	0.643	Age, years	38.0 (34.5–48.5)	38.0 (27.5–48.5)	0.392
Females, *n* (%)	14 (77.8)	11 (68.8)	0.551	Females, *n* (%)	28 (96.6)	27 (93.1)	0.553
Disease features and therapy							
GCA	Results			TA	Results		
Headache, *n* (%)	12 (66.7)			Disease duration, months	108 (60–186)		
Constitutional symptoms, *n* (%)	8 (44.4)			Angiographic type V, *n* (%)	16 (55.2)		
Cranial ischemic manifestations, *n* (%)	8 (44.4)			Previous ischemic events, *n* (%)	11 (37.9)		
Jaw claudication, *n* (%)	6 (33.3)			Active disease, *n* (%)	11 (37.9)		
Visual symptoms, *n* (%)	4 (22.2)			Remission, *n* (%)	18 (62.1)		
Polymyalgia rheumatica, *n* (%)	4 (22.2)			Statins, *n* (%)	16 (55.2)		
Headache, *n* (%)	12 (66.7)			Prednisone, *n* (%)	16 (55.2)		
ESR, mm/1^st^ hour	69.6 ± 28.7			Prednisone daily dose, mg	8.7 (5.0–28.7)		
CRP, mg/l	40.0 (20.2–84.2)			Immunosuppressive agents, *n* (%)	19 (65.5)		
Positive TAB, *n*/total	8/11			Biological agents, *n* (%)	9 (31.0)		
Positive PET-CT scan, *n*/total	13/15						

Continuous variables are presented as mean ± standard deviation or as median and interquartile range

*CRP* C-reactive protein, *ESR* erythrocyte sedimentation rate, *GCA* giant cell arteritis, *HC* healthy controls, *n* number of patients, *PET-CT* positron emission computed tomography, *TA* Takayasu arteritis, *TAB* temporal artery biopsy

Active disease in GCA was considered if patients presented manifestations of active disease (e.g. temporal headache, optic neuritis, jaw claudication) not attributable to other causes and/or polymyalgia rheumatica (PMR) symptoms with an increase in ESR > 30 mm/hour whereas remission was considered in the absence of GCA manifestations with normal ESR [30]. Kerr’s criteria and the Indian Takayasu activity score 2010 (ITAS2010) with acute phase response (ITAS.A) using ESR or CRP were employed to ascertain disease activity in TA [31, 32].

In the 18 GCA patients, blood samples were collected at disease onset prior to glucocorticoid therapy and follow-up samples were obtained from 13 patients at 3 months and from six patients at 12 months. Blood samples were collected from 29 TA patients as a cross-sectional evaluation.

### Serum HMGB1

Serum HMGB1 levels were determined by enzyme-linked immunosorbent assay (ELISA) using a commercial kit (Shino Test Corp., Sagamihara, Kanagawa, Japan) according to the manufacturer’s instructions. Results were expressed in nanograms per milliliter.

### Statistical analysis

Statistical analysis was performed using IBM SPSS software for Windows version 20.0 (IBM Corp, Armonk, NY, USA) and graphs were created with GraphPad Prism version 3.02 (GraphPad Software, La Jolla, CA, USA). Mean ± standard deviation or median and interquartile range were used to present normally distributed and nonnormally distributed continuous variables, respectively. Categorical variables were presented as total number and percentage. Comparisons between groups were performed using Student’s *t* test or Mann–Whitney *U* test for continuous data or using chi-square test or Fisher’s exact test for categorical variables. Correlations between numerical data were performed with Spearman’s correlation coefficient. A linear regression model was built to analyze whether age and the diagnosis of LVV were independently associated with serum HMGB1 levels. Receiver operating characteristic (ROC) analysis was performed to find out the HMGB1 cutoff with the best sensitivity and specificity to differentiate GCA from TA. The cutoff value was chosen from the maximized sum of sensitivity and specificity. Paired *t* test or Wilcoxon’s test were used to analyze longitudinal data. The significance level accepted was 5 % (*p* < 0.05).

## Results

### Disease features and therapy of GCA and TA patients

Disease features and therapy of GCA and TA patients are described in Table 1. After the first evaluation, all GCA patients were treated with high-dose prednisolone (60 mg/day) with slow tapering after improvement of disease symptoms and laboratory abnormalities. Disease relapse was observed in four (22.2 %) GCA patients and the median time to the first relapse after diagnosis was 6.0 months (6.0–15.0). Methotrexate 10–15 mg per week was added to two patients (11.1 %) after the first relapse during steroid tapering. Five GCA patients (27.8 %) were on statins at disease onset.

Previous ischemic events in TA included unstable angina (four patients), stroke (three patients), acute myocardial infarction (two patients), transient ischemic attacks and mesenteric ischemia in one patient each. Two TA patients were treated only with prednisone whereas the remainder used either an immunosuppressive drug or a biologic agent. ESR, ITAS.A ESR and ITAS.A C-reactive protein (CRP) values were significantly higher in TA patients with active disease than in those in remission, whereas there was a trend for higher serum CRP levels in patients with active disease. No significant differences could be found between patients with active disease and remission regarding therapy (Table 2).

**Table 2:** Comparison between patients with Takayasu arteritis with active disease and in remission

Variables	Active disease (*n* = 11)	Remission (*n* = 18)	*p*
HMGB1, ng/ml	1.31 (0.63–2.16)	0.75 (0.39–2.05)	0.281
ESR, mm/1^st^ hour	39.0 (25.0–68.0)	17.5 (8.0–25.5)	0.017
CRP, mg/l	6.0 (4.4–24.9)	2.0 (0.1–10.7)	0.053
ITAS2010	3.0 (2.2–5.2)	--	--
ITAS.A ESR	3.5 (2.0–6.2)	1.0 (1.0–1.7)	0.001
ITAS.A CRP	5.1 ± 2.5	2.1 ± 0.9	0.012
Statins, *n* (%)	7 (63.6)	9 (50.0)	0.702
Prednisone, *n* (%)	6 (54.5)	10 (55.6)	0.958
Prednisone daily dose, mg	20.0 (7.5–45.0)	5.0 (2.5–13.7)	0.055
Immunosuppressive agents, *n* (%)	7 (63.6)	12 (66.7)	0.868
Biological agents, *n* (%)	3 (27.3)	6 (33.3)	0.732

Continuous variables are presented as median and interquartile range or as mean ± standard deviation

*CRP* C-reactive protein, *ESR* erythrocyte sedimentation rate, *ITAS* Indian Takayasu activity score, *ITAS.A* Indian Takayasu activity score with acute phase response, *HMGB1* high mobility group box 1, *n* number of patients

### HMGB1 levels in giant cell arteritis

**Fig. 1:**
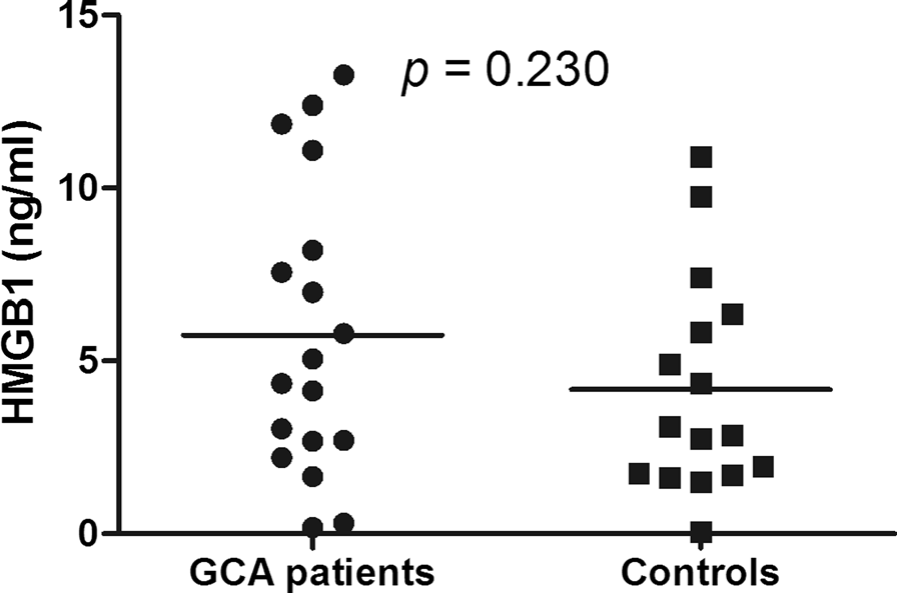
Serum high mobility group box 1 (HMGB1) levels in patients with giant cell arteritis (GCA) and healthy controls (HC). GCA patients at disease onset present similar serum HMGB1 levels compared to HC

In GCA patients with active disease at onset and prior to therapy mean serum HMGB1 levels did not differ between patients and HC (5.74 ± 4.19 ng/ml vs. 4.17 ± 3.14 ng/ml; *p* = 0.230) (Fig. 1). Furthermore, among GCA patients mean serum HMGB1 levels at onset were not higher in patients with or without PMR [1.25 (0.21–10.50) ng/ml vs. 5.42 (2.94–8.92) ng/ml; *p* = 0.167], cranial ischemic manifestations (5.56 ± 3.31 ng/ml vs. 5.89 ± 4.95 ng/ml; *p* = 0.873), constitutional symptoms (4.92 ± 3.90 ng/ml vs. 6.40 ± 4.50 ng/ml; *p* = 0.474) or relapsing disease (4.75 ± 3.31 ng/ml vs. 6.02 ± 4.47 ng/ml; *p* = 0.608), respectively.

**Fig. 2:**
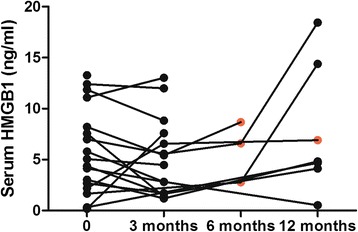
Longitudinal levels of serum high mobility group box 1 (HMGB1) in patients with giant cell arteritis (GCA). Serum HMGB1 in individual GCA patients along follow-up and during relapses (*red dots*)

Mean serum HMGB1 levels in GCA patients were 5.74 ± 4.19 ng/ml at baseline, 5.18 ± 3.98 ng/ml at 3 months, 8.19 ± 6.80 ng/ml at 12 months, and 6.23 ± 2.48 ng/ml at the first relapse. During follow-up, no significant fluctuations on serum HMGB1 levels were observed from baseline levels to 3 and 12 months (Fig. 2). Moreover, serum HMGB1 levels in relapsing patients were not different from their levels at disease onset (*p* = 0.825), at 3 months (*p* = 0.629), at 12 months (*p* = 0.601) and from HC (*p* = 0.170) (Table 3). In GCA patients no correlation was present between HMGB1 and ESR (rho = −0.220; *p* = 0.380) or between HMGB1 and CRP levels (rho = −0.258; *p* = 0.301).

**Table 3:** Longitudinal data on disease activity and serum HMGB1 levels in patients with giant cell arteritis

Variables	Baseline (*n* = 18)	3 months (*n* = 13)	12 months (*n* = 6)	Relapse (*n* = 4)
HMGB1, ng/ml	5.74 ± 4.19	5.18 ± 3.98	8.19 ± 6.80	6.23 ± 2.48
ESR, mm/1^st^ hour	69.6 ± 28.7	15.1 ± 6.6	21.0 ± 4.9	57.5 ± 24.2
CRP, mg/l	40.0 (20.2–84.2)	2.5 (2.5–7.0)	8.0 (5.1–14.7)	38.5 (12.0–82.2)
Prednisolone, mg/day	--	20.0 (18.7–27.5)	18.7 (3.7–30.0)	6.2 (1.2–9.3)

Continuous variables are presented as median and interquartile range or as mean ± standard deviation

*CRP* C-reactive protein, *ESR* erythrocyte sedimentation rate, *HMGB1* high mobility group box 1

### Serum HMGB1 in Takayasu arteritis

**Fig. 3:**
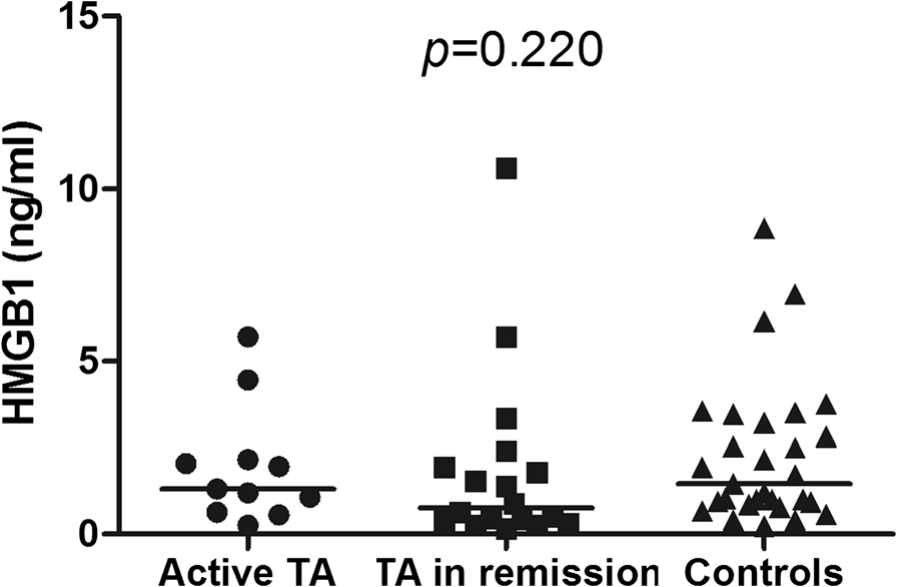
Serum high mobility group box 1 (HMGB1) levels in patients with Takayasu arteritis (TA) and healthy controls (HC). TA patients with active disease and in remission present similar serum HMGB1 levels compared with HC

**Fig. 4:**
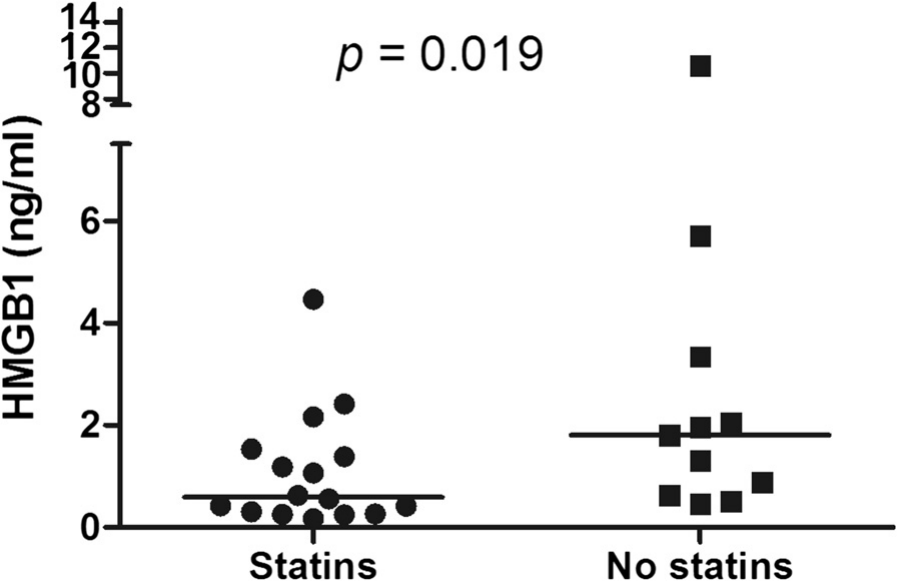
Influence of statins use on serum high mobility group box 1 (HMGB1) levels in patients with Takayasu arteritis (TA). Statins use was associated with significantly lower serum HMGB1 levels in TA patients

As depicted in Fig. 3, serum HMGB1 levels did not differ between TA patients with active disease [1.31 (0.63–2.16) ng/ml], patients in remission [0.75 (0.39–2.05) ng/ml] and HC [1.46 (0.89–3.34) ng/ml] (*p* = 0.220). Similar median serum HMGB1 levels were found in TA patients with and without previous ischemic events [1.53 (0.42–3.34) ng/ml vs. 0.97 (0.50–1.93) ng/ml; *p* = 0.486]. There was no difference in serum HMGB1 levels in TA patients under prednisone therapy compared with those not receiving prednisone [1.13 (0.45–2.34) ng/ml vs. 1.31 (0.36–1.94) ng/ml; *p* = 0.676] or between TA patients receiving immunosuppressive agents compared with those on biological agents [1.59 (0.43–2.45) ng/ml vs. 0.59 (0.42–0.96); *p* = 0.140]. However, serum HMGB1 levels were significantly lower in TA patients on statins compared with patients not receiving these agents [0.59 (0.29–1.46) ng/ml vs. 1.93 (0.88–3.34) ng/ml; *p* = 0.019] (Fig. 4).

No correlation could be observed between serum HMGB1 levels and ESR (rho = 0.104; *p* = 0.590), CRP (rho = 0.090; *p* = 0.642), ITAS2010 (rho = 0.230; *p* = 0.231), ITAS.A ESR (rho = 0.216; *p* = 0.261) or ITAS.A CRP (rho = 0.070; *p* = 0.720).

### Comparison between Takayasu arteritis and giant cell arteritis regarding serum HMGB1 levels

**Fig. 5:**
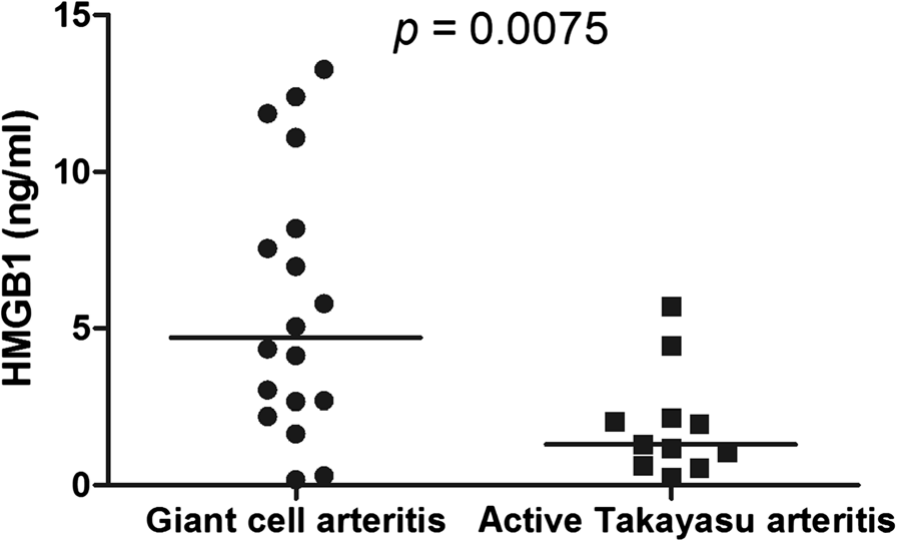
Serum high mobility group box 1 (HMGB1) levels in patients with giant cell arteritis (GCA) and Takayasu arteritis (TA) with active disease. GCA patients at disease onset and prior to any therapy present higher serum HMGB1 levels than TA patients with active disease but already on treatment with prednisone and immunosuppressive or biological agents

GCA patients at disease onset presented significantly higher median serum HMGB1 levels compared with TA patients with active disease [4.70 (2.55–8.92) ng/ml vs. 1.31 (0.63–2.16) ng/ml; *p* = 0.0075] (Fig. 5). Even when GCA and TA patients without statins were analyzed separately, serum HMGB1 levels were significantly higher in GCA patients compared to TA patients [5.06 (2.86–10.0) ng/ml vs. 1.80 (0.63–3.34); *p* = 0.015].

Higher serum HMGB1 levels observed in GCA compared with TA seems to be an effect of aging, since serum HMGB1 levels were also higher in GCA controls than in TA controls [2.98 (1.70–6.23) ng/ml vs. 1.46 (0.89–3.34) ng/ml; *p* = 0.019]. A weak correlation was found between serum HMGB1 levels and age in all study participants (rho = 0.244; *p* = 0.019) while in a linear regression model, age was independently associated with serum HMGB1 levels (β = 0.056; *p* = 0.003; R^2^ = 0.099), regardless of the diagnosis of LVV or control status. ROC analysis of GCA and TA patients showed that the best HMGB1 cutoff value for differentiating GCA from TA is 2.17 ng/ml with 83.3 % sensitivity and 79.3 % specificity.

## Discussion

In this study, we observed that patients with active LVV present similar serum HMGB1 levels compared with patients in remission and HC. TA patients in remission and those with relapsing disease were already under therapy and the use of statins was associated with lower serum HMGB1 levels. Furthermore, in GCA patients with active disease prior to therapy, serum HMGB1 levels were not different from HC but were higher than HMGB1 levels found in TA patients with active disease.

The need for reliable biomarkers for disease activity is an issue of utmost importance in TA. The evaluation of disease activity is a challenge; since the disease course is protracted and silent relapses are common, occurring in up to 96 % of patients who attained remission. It is not easy to define when the disease is actually in remission and most patients develop new angiographic lesions over time usually without clear manifestations of disease activity [33]. In this context, a novel biomarker would help medical decisions for TA.

Granulomatous inflammation and vessel wall necrosis are well-known features of LVV [34]. Either necrosis or infiltrating macrophages are important sources of HMGB1 release into the extracellular milieu that in turn activate innate and adaptive immunity [35]. Patients with GPA and predominant granulomatous inflammation present higher serum HMGB1 levels compared with GPA patients with predominantly vasculitic manifestations [25]. Thus, we evaluated associations between disease activity in LVV and serum HMGB1 levels. Unfortunately, no difference could be found between patients with active disease and remission or between patients with LVV and HC.

On the other hand, GCA patients at disease onset and prior to therapy presented serum HMGB1 levels that were similar to those of HC, and no association could be found between HMGB1 and acute phase reactants, disease manifestations or disease relapse. Moreover, during follow-up no significant fluctuations in serum HMGB1 levels were observed in GCA patients. Novel biomarkers in GCA would help to recognize active disease in patients with signs and symptoms of GCA but normal acute phase reactants. However, serum HMGB1 levels were not increased in patients with active disease.

Serum HMGB1 levels were significantly higher in GCA patients than in TA patients, and even though the ROC analysis showed that a cutoff value of 2.17 ng/ml in HMGB1 levels would help to differentiate GCA from TA, we believe that it is unlikely that in clinical practice it would replace the 50-year-old cutoff point used to differentiate both entities [1]. Furthermore, GCA controls had higher serum HMGB1 than TA controls. These findings indicate that serum HMGB1 levels increase during aging and may be influenced by the burden of atherosclerosis in older individuals. In mice, the age-dependent DNA double-strand break is associated with a reduction of nuclear HMGB1 in neurons leading to an increased release of extracellular HMGB1 [36]. However, in a population study performed in Japan with 626 subjects, aging did not seem to affect serum HMGB1 levels in healthy subjects [37]. In the present study, although only a weak correlation was found between age and serum HMGB1 levels, age was independently associated with serum HMGB1 levels regardless of the diagnosis of LVV or control status.

We found a strong association between statins and lower serum HMGB1 levels in 16 patients with TA (55.2 %). Recently, lower HMGB1 levels were observed in hyperlipidemic patients and in GPA patients in remission both on statin therapy [38, 39]. Moreover, atorvastatin was able to reduce in vitro the release of HMGB1 in stimulated human umbilical vein endothelial cell (HUVEC) cultures. This indicates that the inhibition of HMGB1 release by activated cells is one of the pleiotropic effects of statins [39]. Other drugs may also influence HMGB1 release from cells such as dexamethasone and metformin [40, 41]. These findings may explain in part why TA patients already under treatment presented serum HMGB1 levels similar to HC.

The role of statins in GCA has still to be determined. No impact on relapse rate or on the prevention of severe ischemic events was observed in retrospective studies. However, conflicting results were found regarding the influence of statins on acute phase reactants and daily glucocorticoid dose in GCA patients using statins [42–44]. In TA patients, a retrospective study could not find any difference in ischemic events between patients with and without statins but associations with disease activity were not analyzed [45]. In the present study, more TA patients used statins than GCA patients at diagnosis although this difference was not statistically significant (data not shown). This could be due to the long disease course of our TA patients in comparison with the GCA patients who were evaluated at disease onset.

Limitations of this study are its mainly cross-sectional nature and the inclusion of patients already on therapy for TA, whereas the low number of patients and the short-term follow-up period are limitations for the GCA patients. Nevertheless, the data seem robust enough to conclude that HMGB1 is not a suitable biomarker in LVV in contrast to SLE [23].

## Conclusions

Serum HMGB1 levels were neither different between patients with LVV and HC, nor between patients with active disease and those in remission. Therefore, serum HMGB1 is not a useful biomarker for LVV. Moreover, serum HMGB1 levels were not associated with any disease phenotypes in LVV. In long-standing TA, therapy with statins seems to lead to lower serum HMGB1 levels.

## Abbreviations

18FDG-PET/CT: 18^F^-fluorodeoxyglucose positron emission computed tomography
ACR: American College of Rheumatology
ANCA: antineutrophil cytoplasmic antibody
BAFF: B cell-activating factor
CRP: C-reactive protein
CXCL9: chemokine (C-X-C motif) ligand 9
ELISA: enzyme-linked immunosorbent assay
ESR: erythrocyte sedimentation rate
GCA: giant cell arteritis
GPA: granulomatosis with polyangiitis
HC: healthy controls
HMGB1: high mobility group box 1
HUVEC: human umbilical vein endothelial cell
IFN: interferon
Ig: immunoglobulin
IL: interleukin
ITAS: Indian Takayasu activity score
ITAS.A: ITAS with acute phase response
LPS: lipopolysaccharide
LVV: large vessel vasculitides
MCP-1: monocyte chemoattractant protein-1
MMP-9: matrix metalloproteinase 9
PMR: polymyalgia rheumatica
RANTES: regulated on activation, normal T cell expressed and secreted
ROC: receiver operating characteristic
SLE: systemic lupus erythematosus
TA: Takayasu arteritis
Th: T helper cell
TNF-α: tumor necrosis factor alpha
UMCG: University Medical Center Groningen
UNIFESP: Universidade Federal de São Paulo
